# Predicting Where a Radiation Will Occur: Acoustic and Molecular Surveys Reveal Overlooked Diversity in Indian Ocean Island Crickets (Mogoplistinae: *Ornebius*)

**DOI:** 10.1371/journal.pone.0148971

**Published:** 2016-02-12

**Authors:** Ben H. Warren, Rémy Baudin, Antoine Franck, Sylvain Hugel, Dominique Strasberg

**Affiliations:** 1 Université de La Réunion, UMR PVBMT, 7 chemin de l’IRAT, Ligne Paradis, 97410 Saint Pierre, Réunion, France; 2 Institute of Systematic Botany, University of Zurich, Zollikerstrasse 107, 8008, Zurich, Switzerland; 3 Cirad, UMR PVBMT, 7 chemin de l’IRAT, Ligne Paradis, 97410 Saint Pierre, Réunion, France; 4 UPR 3212 CNRS, Université de Strasbourg, 5 rue Blaise Pascal, 67084 Strasbourg, cedex 3, France; University of Massachusetts, UNITED STATES

## Abstract

Recent theory suggests that the geographic location of island radiations (local accumulation of species diversity due to cladogenesis) can be predicted based on island area and isolation. Crickets are a suitable group for testing these predictions, as they show both the ability to reach some of the most isolated islands in the world, and to speciate at small spatial scales. Despite substantial song variation between closely related species in many island cricket lineages worldwide, to date this characteristic has not received attention in the western Indian Ocean islands; existing species descriptions are based on morphology alone. Here we use a combination of acoustics and DNA sequencing to survey these islands for *Ornebius* crickets. We uncover a small but previously unknown radiation in the Mascarenes, constituting a three-fold increase in the *Ornebius* species diversity of this archipelago (from two to six species). A further new species is detected in the Comoros. Although double archipelago colonisation is the best explanation for species diversity in the Seychelles, in situ cladogenesis is the best explanation for the six species in the Mascarenes and two species of the Comoros. Whether the radiation of Mascarene *Ornebius* results from intra- or purely inter- island speciation cannot be determined on the basis of the phylogenetic data alone. However, the existence of genetic, song and ecological divergence at the intra-island scale is suggestive of an intra-island speciation scenario in which ecological and mating traits diverge hand-in-hand. Our results suggest that the geographic location of *Ornebius* radiations is partially but not fully explained by island area and isolation. A notable anomaly is Madagascar, where our surveys are consistent with existing accounts in finding no *Ornebius* species present. Possible explanations are discussed, invoking ecological differences between species and differences in environmental history between islands.

## Introduction

Species may be added to communities by two different processes; either they arrive by immigration from outside, or they are formed in situ by speciation. MacArthur and Wilson [1] were among the first to make quantitative predictions regarding the influence of immigration in determining local species richness. In their equilibrium theory of island biogeography, immigration rates are expected to decrease with island isolation with ultimate species richness resulting from an equilibrium between immigration and extinction. It was later noted that larger islands have a larger interception area (or shoreline) implying a higher immigration rate than for smaller islands [2,3]. MacArthur and Wilson’s core model (Core IBT [4]) did not, however, include speciation, despite the authors’ recognition of its importance in determining species richness on sufficiently isolated islands in what they termed a ‘radiation zone’. More recently, the role of higher speciation rates in larger areas has been incorporated into predictions of how species richness varies with island area [5–9]. Rosindell and Phillimore [10] consider the geographic modes of speciation on islands, noting that island populations may diverge from mainland populations (speciation by anagenesis) at intermediate levels of isolation, but also from insular sister populations (speciation by cladogenesis) at higher levels of isolation. Their model makes predictions regarding the position of the zone of radiation with respect to island area and isolation, under the assumption that all species are ecologically equivalent.

Crickets (Grylloidea) are an excellent group for testing our ability to predict the position of the radiation zone with respect to island area and isolation. On the one hand, they have high dispersal abilities and have reached some of the most remote oceanic archipelagos worldwide; for example they are one of only two Orthopteran superfamilies to have reached the Hawaiian archipelago without human intervention [11]. On the other hand, unlike many other groups with such high dispersal abilities, crickets are believed to be capable of speciating at small spatial scales and low levels of geographic isolation. Although many island cricket radiations are proposed, few are supported by molecular analyses. Among the limited number for which phylogeographic studies have been conducted, a plethora of intra-island speciation has been documented [12,13]. The most extensively studied case concerns the four largest Hawaiian islands, each of which have hosted the intra-island cladogenetic speciation of between four and six endemic species in the genus *Laupala*. Such closely related species are morphologically cryptic, dietary generalists, with no ecologically distinguishable features. However, they can be distinguished based on the pulse rate of male courtship song, and it appears that divergence in such secondary sexual traits has been key in their intra-island cladogenetic speciation [12,14], allowing gene flow to be interrupted within small geographic areas despite high dispersal abilities.

In contrast to the situation in the Hawaiian islands, in the islands of the western Indian Ocean, the predominant known cases of closely related co-occurring cricket species involve species with high levels of ecological and morphological segregation. For example, of the two *Ornebius* (flightless Grylloidea, Mogoplistinae) species known to co-occur on islands of the Granitic Seychelles, one species–*Ornebius validus*–is found exclusively in coastal habitat, while the other–*Ornebius elegantulus*–is restricted to the higher-level forests in the interior of the islands. The high degree of morphological divergence between these two species relative to that seen in the genus as a whole (in terms of the head, thorax, wings, genitalia and overall colouration) [15] is strongly indicative of their co-occurrence resulting from two independent colonisations of the Granitic Seychelles archipelago, rather than intra-island (or intra-archipelago) speciation.

**Fig 1:**
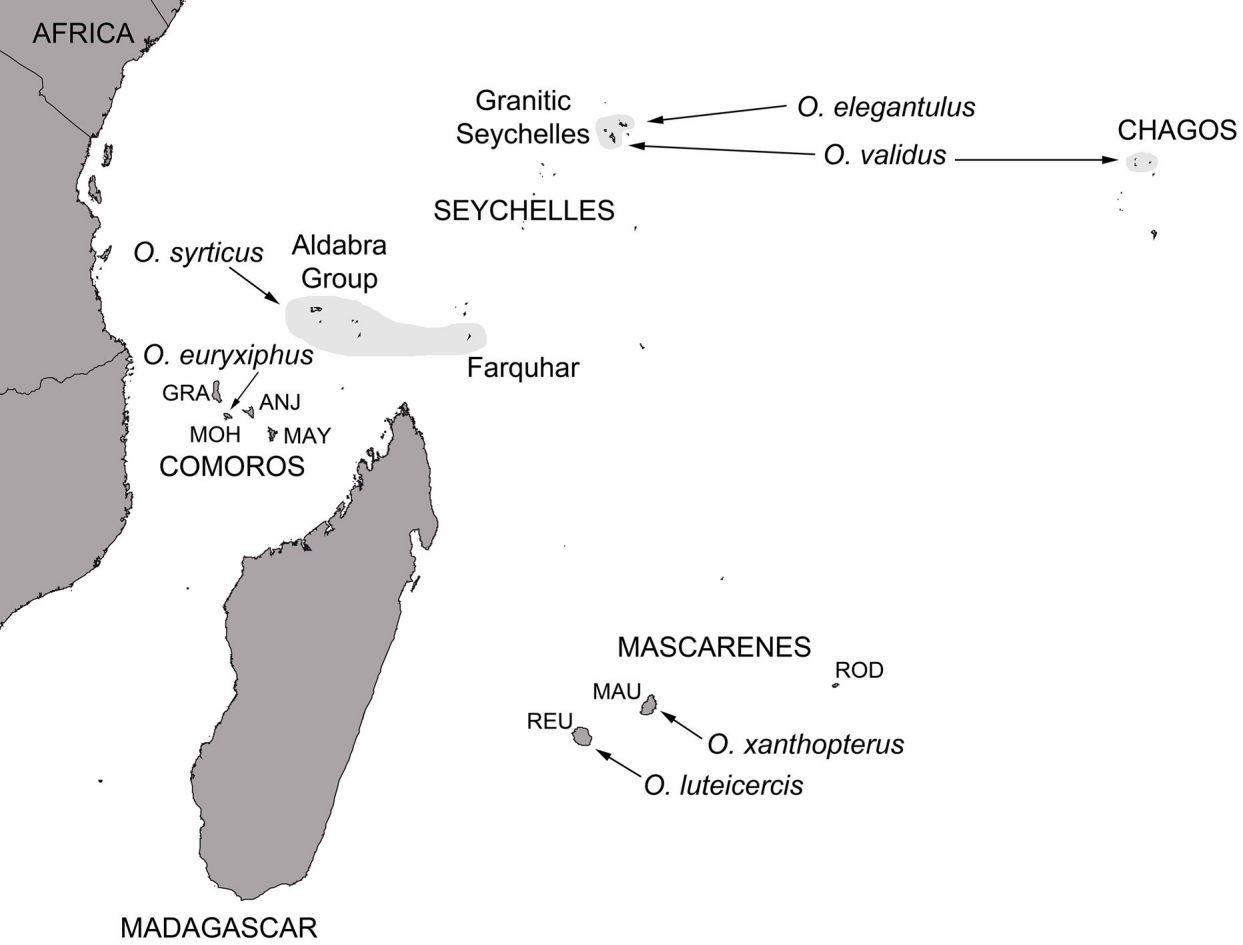
The documented distribution of described *Ornebius* species of the western Indian Ocean. Species distributions across multiple small islands are indicated with pale grey shading. Synonyms are excluded. REU, Réunion; MAU, Mauritius; ROD, Rodrigues; GRA, Grande Comore; MOH, Moheli; ANJ, Anjouan; MAY, Mayotte.

Only four other *Ornebius* species are known from the islands of the western Indian Ocean (*Ornebius xanthopterus* from Mauritius, *Ornebius luteicercis* from Réunion, *Ornebius syrticus* from the Aldabra Group and Farquhar, and *Ornebius euryxiphus* from Mohéli; Fig 1). Although males are highly vocal in attracting females, song divergence was apparently not used in species discoveries, and descriptions are based on morphology alone [15–19].

Despite the importance of morphological data in taxonomy, in the past decade, cryptic species have been found to be common among invertebrates (both on islands and continents [12,20]). Crickets are a case in point, in which acoustic data have proven particularly valuable in uncovering cryptic diversity [12,21]. In light of these discoveries, we use acoustics to survey the islands of the western Indian Ocean for *Ornebius* crickets, using sound both to locate individuals and to identify putatively divergent populations. We then construct a molecular phylogenetic hypothesis for all such putatively divergent populations encountered. Levels of genetic divergence are used to assign undescribed lineages to candidate species, based on the minimum genetic divergence between described species. Formal description of the new species including morphological data will be made elsewhere (Hugel et al. unpublished data). We seek to determine whether the distribution of richness in anagenetic and cladogenetic species conforms with predictions from Rosindell and Phillimore’s [10] model, considering island size and isolation. Based on the apparent situation in the Granitic Seychelles, we test the default hypothesis that all cases of multiple *Ornebius* taxa occupying Indian Ocean archipelagos result from a corresponding number of colonisations from outside the archipelago (and potentially anagenesis), rather than in situ speciation (cladogenesis).

## Methods

### Regional diversity discovery: acoustic and museum surveys

Given the significant volume of unidentified Ensifera from Madagascar in museums, initial surveys for undocumented *Ornebius* diversity were made by examining unidentified material from the Muséum National d’Histoire Naturelle (Paris) and the Natural History Museum (London). Permission to access these collections was granted by the curators (L. Desutter-Grandcolas and G. Beccaloni, respectively). Material from both museums was examined directly on site (and was therefore not loaned, purchased, nor donated). The Paris museum houses a large collection that was formerly part of the Institut Scientifique de Madagascar.

Acoustic surveys were performed by SH during the last 10 years, in the process of compiling an inventory of the orthopteroid fauna of islands around Madagascar. The islands visited were the following: Comoros: 48 survey nights on Grande Comore, Anjouan, Mohéli, Mayotte (4/2010, 8/2010, 12/2011); Seychelles: 25 survey nights on Mahé, La Digue, Praslin, Silhouette (7/2003, 8/2010); Madagascar: 20 survey nights in the regions of Antananarivo, Fianarantsoa, Toamasina, Toliara (1/2011); Rodrigues: 43 survey nights (4/2005 (5); 2/2008 (5); 4/2009 (5); 4/2011 (5), 4/2012 (5), 10/2013 (10), 7/2015 (8)); Réunion: 99 survey nights (6/2002 (10), 4/2004 (8), 12/2005 (10), 5/2007 (8), 11/2009 (20), 8/2012 (20), 2/2013 (15), 2/2014 (10), 5/2015 (8)); Mauritius: 79 survey nights (7/2002 (10), 5/2005 (5), 3/2008 (18), 4/2009 (15), 5/2011 (5), 5/2012 (8), 10/2013 (10), 8/2015 (8)).

In all these localities, for every survey night at least four hours were spent in the field collecting insects by sound and sight, with a headlamp. We found that the frequency of Indian Ocean *Ornebius* song (always within the 4–7 kHz range) falls well within the human acoustic range (approximately 0.02–16 kHz). Therefore most crickets were initially located by ear (i.e. without stereo equipment), either by moving around the apparent sound source in inward spirals, or using two or more people to locate each sound source by approximate triangulation. Once an individual was located, song was recorded with an Audiotechnica AT822 stereo microphone (ca. flat response 200 Hz—20 kHz) on a HDR HC1E Sony camcorder (sampling rate: 48 kHz). A camera set to nightshot (infrared) mode was used to ensure the correspondence between sound recordings and the individual located. Where possible, the recorded specimen was captured and a hind leg was preserved in absolute ethanol. If captured individuals were not recorded in the field or not visible during the field recording, these were kept alive and subsequently recorded under studio conditions. As *Ornebius* duetting song (i.e. song produced by two or more interacting males) is faster than calling and courtship songs (SH unpublished data), all studio recordings considered here were from males kept alone or with females, never with other males. Where possible, on each island, at least three individuals per song type were recorded.

**Fig 2:**
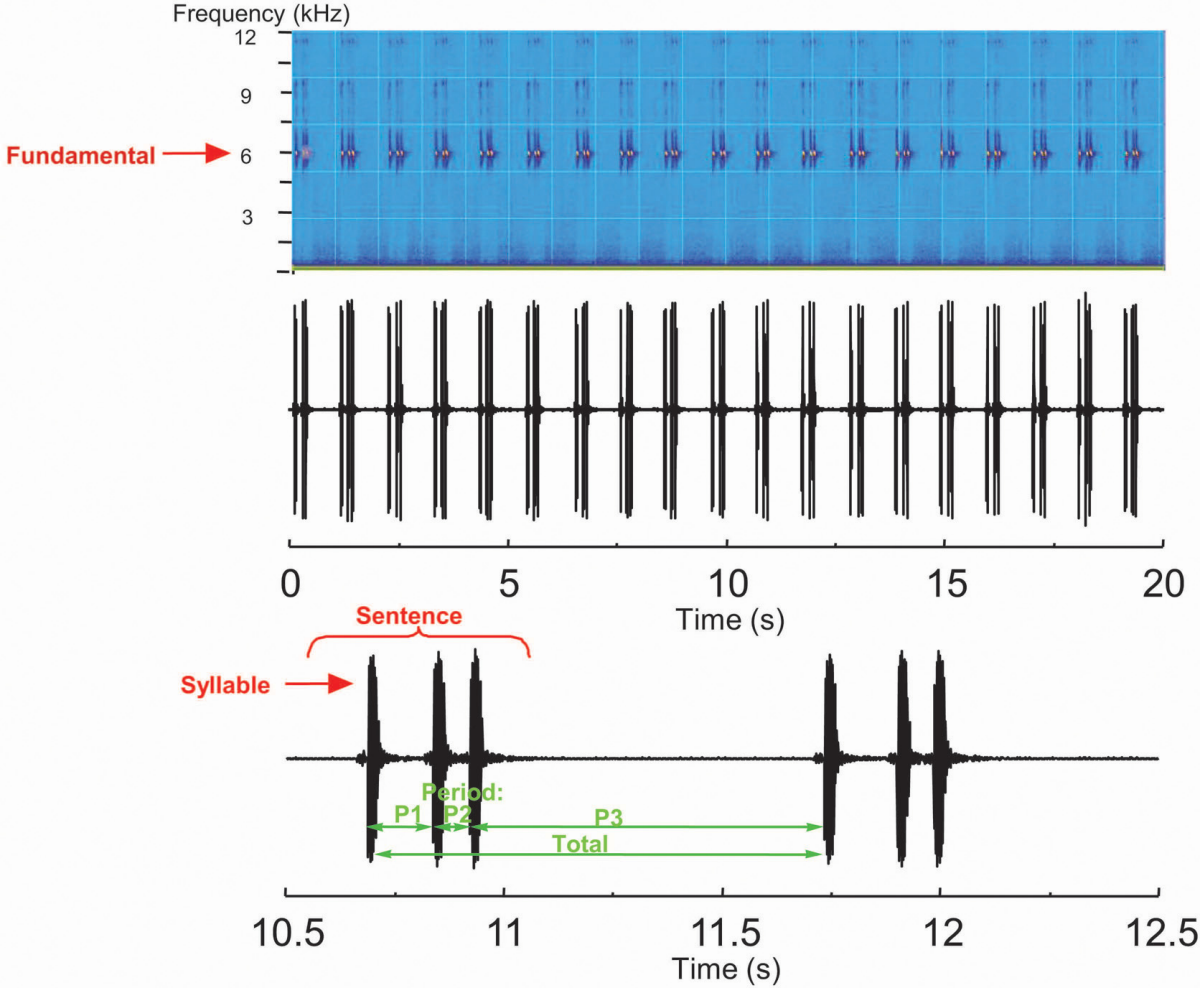
Measurements taken from recorded *Ornebius* songs. The fundamental (frequency of the first harmonic); pattern of the song sentence (mono-, di-, tri-syllabic); period of the first syllable (P1); period of the second syllable (P2; where applicable); period of the third syllable (P3; where applicable); period of sentence repetition (Total).

In order to standardise for temperature within available field conditions, all recordings were restricted to temperatures within the range of 20 to 29°C. Depending on constraints of field conditions, between two and four individuals were recorded at two or three different temperatures, and their frequency, pulse rate and sentence duration were used to determine 22°C equivalents using a standard curve fitted using a linear function. All individuals recorded at temperatures other than 22°C were corrected to this 22°C equivalent, treating the effects of temperature on song (frequency, pulse rate and sentence duration) as consistent across all species considered. Accordingly, the first harmonic was increased by 120 Hz.°C^-1^, the pulse rate was increased by 0.4°C^-1^, and the sentence duration by 2.5%°C^-1^. Song terminology is after Ragge and Reynolds [22]. Songs were analysed using Clampfit 10.2. Six elements of the recorded songs were measured (Fig 2): frequency of the first harmonic (i.e. fundamental); pattern of the song sentence (mono-, di-, tri-syllabic); period of the first syllable; period of the second syllable (where applicable); period of the third syllable (where applicable); period of sentence repetition. In order to classify the song types, the parameters obtained (S1 Table) were analyzed using the K-means classification algorithm implemented in Tangara 1.4. This technique enables data grouping using an algorithm that assigns objects to clusters minimizing the sum of distances from each object to its cluster centroid. The song types of each pattern (i.e. mono-, di-, tri-syllabic) identified by ear in the field were compared to the natural groups given by this algorithm. All work was performed with permits from the relevant authorities: Mauritius: National Park and Forestry Service; Rodrigues: National Park and Forestry Service and Rodrigues Regional Assembly; La Réunion: Office National des Forêts, Parc National de La Réunion; Seychelles: Seychelles Bureau of Standards; Madagascar: Ministère de l’Environnement et des Forêts; Comoros: Centre National de Documentation et de Recherche Scientifique and University of Comoros; Mayotte: Direction de l'Environnement, de l'Aménagement et du Logement.

### Construction of the molecular dataset: regional and continental sampling

Sample sizes were restricted because several Indian Ocean Orthoptera species are threatened [23,24], and in view of conservation priorities, research permits restrict sampling for island endemics with small populations. Further, many species occur in treetops and are highly time-consuming to collect. Depending on numbers of individuals available, between two and four individuals from each natural song group in the western Indian Ocean were sampled from across that group’s range (be it restricted to one island, or found across many), and including at least one individual per island.

*Ornebius* has a wide distribution across the tropics, with most described species occurring in Australasian and Oriental regions, and a minority of occurrences in the Americas and islands of the Pacific and Indian Ocean [25]. A single species is described from Africa (*Ornebius acutus* from Ghana), but probably belongs to a distinct genus (S. Hugel, unpublished data). We therefore obtained *Ornebius* samples from Australia, French Polynesia, Borneo and Central America as potential source areas for the colonisation of the western Indian Ocean, as well as outgroup samples from two other Mogoplistinae genera (*Ectatoderus* and *Cycloptiloides*) and one other Grylloidea (*Creolandreva*).

Each individual was vouchered and stored in Ethanol. DNA was extracted from finely sliced leg tissue using a DNeasy Blood and Tissue Kit (Qiagen Inc., Valencia, CA, USA), and following the manufacturer’s protocol. For all samples (i.e. 2–4 individuals per song group in the Indian Ocean, and 1 individual per continental species), a total of 1594 bp of sequence data were obtained from the mitochondrial COI gene, and ribosomal RNA genes 12S and 16S. Primers used for amplification and sequencing of these three genes were LCO1490 and HCO2198 [26], SR-J-14233 and SR-N-14588 [27], and 16Sa and 16Sb [28], respectively. The polymerase chain reaction (PCR) program began with denaturation for 5 min at 94°C, followed by 35 cycles of 1 min at 94°C, 1 min at 49°C, 1 min at 72°C, and a final extension step at 72°C for 5 min. To guard against the possibility of introgression obscuring true lineage history and improve the resolution of phylogenetic relationships, the mitochondrial data were supplemented with nuclear EF1α and H3 sequences from a reduced sample set (1–3 individuals from each terminal monophyletic group in the mitochondrial dataset), providing a five-gene dataset totalling 2352 bp. Primers used for amplification and sequencing of EF1α and H3 were M51F and M53R [29], and 5’-ATGGCTCGTACCAAGCAGACVGC-3’ and 5’-ATATCCTTRGGCATRATRGTGAC-3’ [30], respectively. The PCR program began with denaturation for 4 min at 94°C, followed by 40 cycles of 30 sec at 94°C, 40 sec at 54°C, and 40 sec at 72°C, and a final extension step at 72°C for 7 min. Amplified products were purified and sequenced by Macrogen (Seoul, Korea) using the standard-seq single service. Sequences have been deposited in Genbank (Accession numbers KC494774-KC494868, KU597648-KU597710).

### DNA partition, model selection and phylogenetic inference

#### Mitochondrial data for the full sample set

The congruence of the different gene data sets was checked using the partition homogeneity test [31] implemented in PAUP*, and data from the three genes were combined for further analysis. The combined dataset excluding outgroups (*Creolandreva*, *Ectatoderus* and *Cycloptiloides*) was analysed in MODELTEST [32] and MrModeltest [33]. The optimal model defined by MODELTEST was used to determine the maximum-likelihood (ML) distances for a ML analysis implemented in PAUP*. In addition, an unweighted parsimony analysis was performed with the heuristic search algorithm, holding 10 trees at each step and branch swapping on all trees.

The optimal model defined by MrModeltest was used for Bayesian analysis implemented in MrBayes 3.2.1 [34]. Base frequencies were estimated from the data. Four Markov chains were run simultaneously for 20 million generations, and sampled every 100 generations. Variation in likelihoods was examined graphically. The trees generated prior to stationarity were discarded, and the consensus phylogeny and posterior probability of its nodes were determined from the last 150,000 trees in the chain. To check our results and guard against the possibility of multiple optima, we repeated this process four times.

#### Nuclear & mitochondrial data for the restricted sample set

The congruence of the different gene data sets was checked using the partition homogeneity test [31] implemented in PAUP*, and data from the five genes were combined for further analysis. An unweighted parsimony analysis was performed as for the mitochondrial dataset. In addition, the combined dataset excluding outgroup (*Ectatoderus*) was analysed in MODELTEST [32] and MrModeltest [33]. The optimal model defined by MrModeltest was used for Bayesian analysis following the procedures for the mitochondrial dataset. The first three analyses were run for 20 million generations, and the consensus phylogeny and posterior probability of its nodes were determined from the last 150,000 trees in the chain. The final analysis was run for 50 million generations and the last 375,000 trees were used to obtain the consensus.

The method of Shimodaira and Hasegawa [35] (SH) implemented in PAUP* was used to test the monophyly of *Ornebius* species and discriminate between alternative scenarios explaining archipelago diversity. Were all endemic taxa of an archipelago a result of a single colonisation event and in situ cladogenesis, we would expect them to be monophyletic (Scenario 1). By contrast, under our default expectation in which each taxon results from an independent colonisation event and anagenesis, they would be paraphyletic (Scenario 2; i.e. archipelago-endemic taxa would be more closely related to lineages from outside the archipelago than they are to each other). The optimal NJ tree constructed with the ML substitution model defined by MODELTEST was compared to equivalent trees constrained to correspond with alternative hypotheses.

## Results

### Acoustic surveys

**Fig 3:**
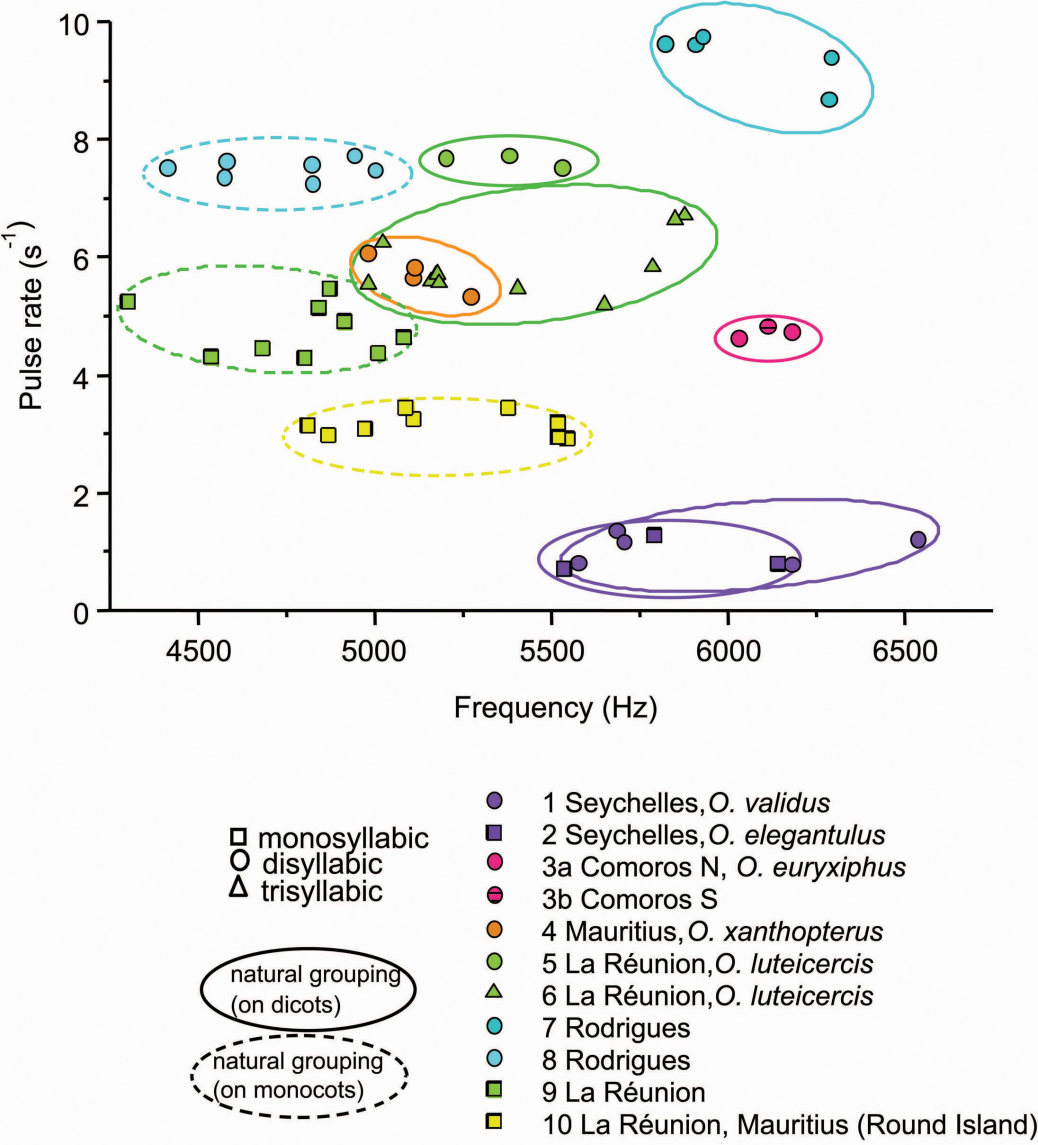
Characteristics of the ten *Ornebius* song types recorded on islands of the western Indian Ocean. Each song type is represented by at least three different individuals. In order to standardise for temperature within the constraints of field conditions, all recordings are restricted to temperatures within the range of 20 to 29°C. Frequency and pulse rate were corrected to a 22°C equivalent using a standard curve (see methods). For di- and tri-syllabic song patterns, the pulse rate of the closest syllables of the echeme is given.

For each song pattern (mono-, di-, and tri-syllabic), distinct song types detected by ear correspond to natural groups obtained by the K-means classification algorithm (Fig 3; data in S1 File). Four of the five described *Ornebius* species from islands in the western Indian Ocean that we were able to visit–*O*. *xanthopterus*, *O*. *elegantulus*, *O*. *validus*, and *O*. *euryxiphus–*each correspond to single natural song groups (Fig 3). Song type 3 (*O*. *euryxiphus)* was recorded on Mohéli, Grande Comore, Anjouan and Mayotte, the latter three islands being new records for *Ornebius* (Table 1). Within *O*. *luteicercis*, two natural song groups occur; a disyllabic song (type 5) encountered only in the northwest quarter of Réunion, and a trisyllabic song (type 6) encountered elsewhere on the island (Fig 3; Table 1). Four other song groups were uncovered in the western Indian Ocean (Fig 3; Table 1). Based on our surveys, each one of these has a restricted distribution; types 7 and 8 being restricted to Rodrigues, type 9 to Réunion, and type 10 to Réunion and islets north of Mauritius (Round Island, Gunner’s Quoin and Gabriel Islet).

**Table 1:** Distribution and host plants of *Ornebius* song types in the western Indian Ocean.

Song type	Distribution of our recordings	Altitudinal distribution	Host plants	Corresponding species	Documented geographic distribution*
1	Granitic Seychelles (La Digue, Praslin, Cousin)	Coastal habitat	Dicots	*O*. *validus* (Bolívar, 1895)	Granitic Seychelles (Mahé, Praslin, Silhouette, Anonyme), Chagos (Salomon, Peros Banhos, Diego Garcia)
2	Granitic Seychelles (Mahé, Praslin, La Digue, Silhouette)	Higher level interior forests	Dicots	*O*. *elegantulus* Bolívar, 1912	Granitic Seychelles (Mahé, Praslin, Silhouette, Cerf, Longue, Curieuse, Fregate)
3†	Comoros archipelago (Grande Comore, Moheli)	Coastal habitat to 800 m	Dicots	*O*. *euryxiphus* Chopard, 1958 (3a)	Moheli
3†	Comoros archipelago (Anjouan, Mayotte)	Coastal habitat to 800 m	Dicots	Unknown (3b)	-
4	Mauritius (mainland only)	Coastal habitat to 700 m	Dicots	*O*. *xanthopterus* Guérin-Méneville, 1844	Mauritius
5	Réunion—NW quarter of island	≤ 800 m	Dicots	*O*. *luteicercis* Chopard, 1957	Réunion
6	Réunion—SE three quarters of island	≤ 800 m	Dicots	*O*. *luteicercis* Chopard, 1957	Réunion
7	Rodrigues	From the coast to island summit	Dicots	Unknown	-
8	Rodrigues	From the coast to island summit	*Pandanus utilis*, *Pandanus heterocarpus*	Unknown	-
9	Réunion	Mostly above 800 m	*Pandanus*, especially *P*. *purpurascens*	Unknown	-
10	Réunion & offshore islets of Mauritius (Round Island, Gunner's Quoin, Gabriel Islet)	Réunion: ≤ 50 m, Round Island: ≤ 300 m	Réunion: *Pandanus utilis*, Round Island: *Pandanus vandermeerschii*	Unknown	-

*Includes distribution of synonyms, and unpublished records from museum collections.

†A single song pattern (3) is detected in the Comoros, corresponding to two species (3a & 3b) based on levels of genetic divergence.

Individuals of song types 8, 9 and 10 are exclusively found on endemic monocots–mostly screw pines (genus *Pandanus*) and more rarely on palm trees–whereas all other song types are restricted to dicotyledonous plants. Of the *Pandanus*-restricted song types, number 8 occurs on both *Pandanus utilis* and *Pandanus heterocarpus* present on Rodrigues. Song type 9 is restricted to Réunion’s montane or submontane rainforest *Pandanus*, and is principally found on *Pandanus purpurascens*. Compatible with the distribution of this habitat, it mostly occurs above 800 m, but descends to 300 m at Mare Longue in the humid east. Song type 10 is restricted to coastal *Pandanus* habitat. On Réunion this habitat consists of *Pandanus utilis*, and rarely exceeds 50 m in altitude, while on Round Island in the absence of any montane forest, it rises to 300 m and consists of *Pandanus vandermeerschii*. Therefore the only case of two *Pandanus*-restricted song types on the same island is on Réunion, where the two groups are segregated by habitat (which is in turn segregated by altitude and humidity), and at least in the current human-modified landscape, the two never appear to come into contact. No associations of *Ornebius* with *Pandanus* were detected outside of the Mascarenes, despite significant surveying in *Pandanus* habitats.

Consistent with the absence of *Ornebius* records from Madagascar [25], we did not uncover any *Ornebius* specimens or song types from this island, despite intensive museum and acoustic field surveys. Our museum survey included > 20 000 Ensifera specimens from Madagascar collected over three centuries in all the main habitat zones [36]. Our fieldwork included 20 acoustic survey nights across the Antananarivo, Fianarantsoa, Toamasina, and Toliara regions of Madagascar, and paying special attention to *Pandanus* habitats.

### Phylogenetic analysis

#### Mitochondrial data for the full sample set

A partition homogeneity test on the combined data [31] (three partitions; 1594 bp) indicated that the COI, 12S and 16S regions did not differ significantly (P = 0.28). We therefore combined the three data sets for further analysis. An unweighted parsimony analysis resulted in 180 most parsimonious trees with a step length of 2633 (CI 0.475, RI 0.720, RC 0.342, HI 0.525). Both MODELTEST and MrModelTest identified the general time reversible (GTR) model of DNA substitution with invariable sites and gamma shape parameter (GTR+I+G) as best describing the data under the Akaike information criterion.

**Fig 4:**
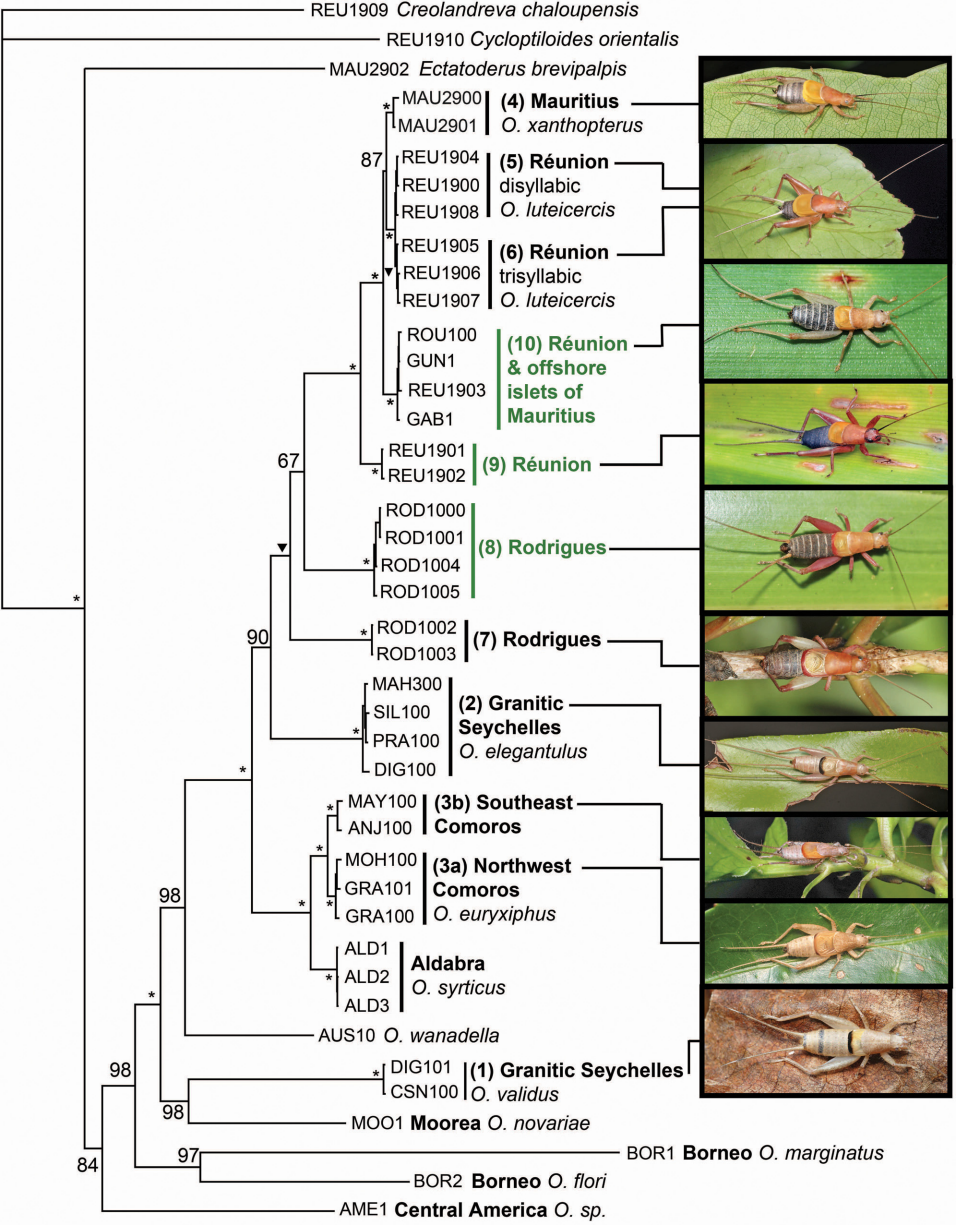
Bayesian analysis of the *Ornebius* mitochondrial three-gene dataset. Consensus of the last 150,000 trees after 20 million generations, based on the GTR+I+G model. Nodes gaining 100% Bayesian posterior probability (PP) are asterisked. Nodes gaining 99% PP are labelled with a triangle, while those gaining 50–98% PP are labelled accordingly. Lineage numbers correspond to song types as listed in Table 1. Lineages labelled in green are associated with *Pandanus*, while those in black are associated with dicots. The islands in which individuals are sampled are coded as in Fig 1; REU, Réunion; MAU, Mauritius; ROD, Rodrigues; GRA, Grande Comore; MOH, Moheli; ANJ, Anjouan; MAY, Mayotte. Islands too small and numerous to label on Fig 1 are as follows. Islands of the Granitic Seychelles: MAH, Mahé; SIL, Silhouette; PRA, Praslin; DIG, La Digue; CSN, Cousin. Islands of the Aldabra Group: ALD, Aldabra Atoll. Islands close to Mauritius: ROU, Round Island; GUN, Gunner’s Quoin; GAB, Gabriel Islet.

Putting aside *O*. *luteicercis* intra-specific song variation, all three methods of phylogenetic reconstruction (maximum parsimony, ML and Bayesian) unanimously support individuals from different song types belonging to highly-diverged monophyletic lineages (each gaining 100% Bayesian posterior probability [PP]; Fig 4). Of these lineages, five correspond to the five known species (*O*. *xanthopterus*, *O*. *luteicercis*, *O*. *elegantulus*, *O*. *validus* and *O*. *euryxiphus*). The song of *O*. *syrticus* has not been recorded, but it is also resolved as monophyletic (100% PP), and divergent from other species. The other four lineages correspond to the other song types 7, 8, 9 and 10. Since the minimum divergence of these latter four undescribed lineages from their closest relatives (whether described or undescribed; 18 substitutions, 1.1% absolute divergence between song type 10 and *O*. *luteicercis*) exceeds the minimum divergence between described species (14 substitutions, 0.88% absolute divergence between *O*. *xanthopterus* and *O*. *luteicercis*), we consider them herein as putative separate species. Divergence within the Comoro lineage corresponding to song type 3 is even higher– 41 substitutions, 2.6% minimum absolute divergence between the Northwest (Grande Comore & Moheli) and Southeast (Anjouan & Mayotte) populations. Following the same logic, we herein consider them as putative separate species, 3a (*O*. *euryxiphus*) and 3b, respectively. The maximum divergence between western Indian Ocean lineages is 292 substitutions (18.3% absolute divergence between *O*. *validus* and species 10), marginally exceeding the maximum divergence between described western Indian Ocean species of 288 substitutions (18.1% absolute divergence between *O*. *validus* and *O*. *luteicercis*).

We consider nodes gaining ≥ 95% Bayesian posterior probability (PP) to be significantly supported [37]. The four Bayesian analyses as well as ML and MP methods yield identical tree topology with respect to such supported nodes between Indian Ocean lineages in the Bayesian trees. All western Indian Ocean lineages form a monophyletic group (100% PP) with the exception of *O*. *validus* which is divergent from the main western Indian Ocean clade, and is sister to *O*. *novariae* in the Bayesian trees (98% PP). Within the western Indian Ocean clade, *O*. *xanthopterus*, *O*. *luteicercis*, and species 7, 8, 9 and 10 form a monophyletic Mascarene-endemic clade with 99% PP. However, within this clade, the *Pandanus*-associated lineages are not monophyletic. Outside the Mascarene clade, the two species present in the Comoros–species 3a (*Ornebius euryxiphus)* and 3b –are monophyletic (100% PP), and sister to *O*. *syrticus* of Aldabra (100% PP).

All *O*. *luteicercis* samples form a monophyletic group with 100% PP. Based on the samples available, the two *O*. *luteicercis* song types (di- and tri- syllabic) appear as separate lineages. However, their divergence is very low (1–6 substitutions, 0.06–0.38% absolute divergence) and only the monophyly of the trisyllabic song type is significantly supported (99% PP). We herein consider *O*. *luteicercis* as a single species pending further data.

#### Nuclear & mitochondrial data for the restricted sample set

A partition homogeneity test on the combined data [31] (five partitions; 2352 bp) indicated that the EF1α, H3, COI, 12S and 16S regions did not differ significantly (P = 0.78). We therefore combined the five data sets for further analysis. An unweighted parsimony analysis resulted in 3 most parsimonious trees with a step length of 1855 (CI 0.644, RI 0.655, RC 0.422, HI 0.356). Both MODELTEST and MrModelTest identified the general time reversible (GTR) model of DNA substitution with invariable sites and gamma shape parameter (GTR+I+G) as best describing the data under the Akaike information criterion.

**Fig 5:**
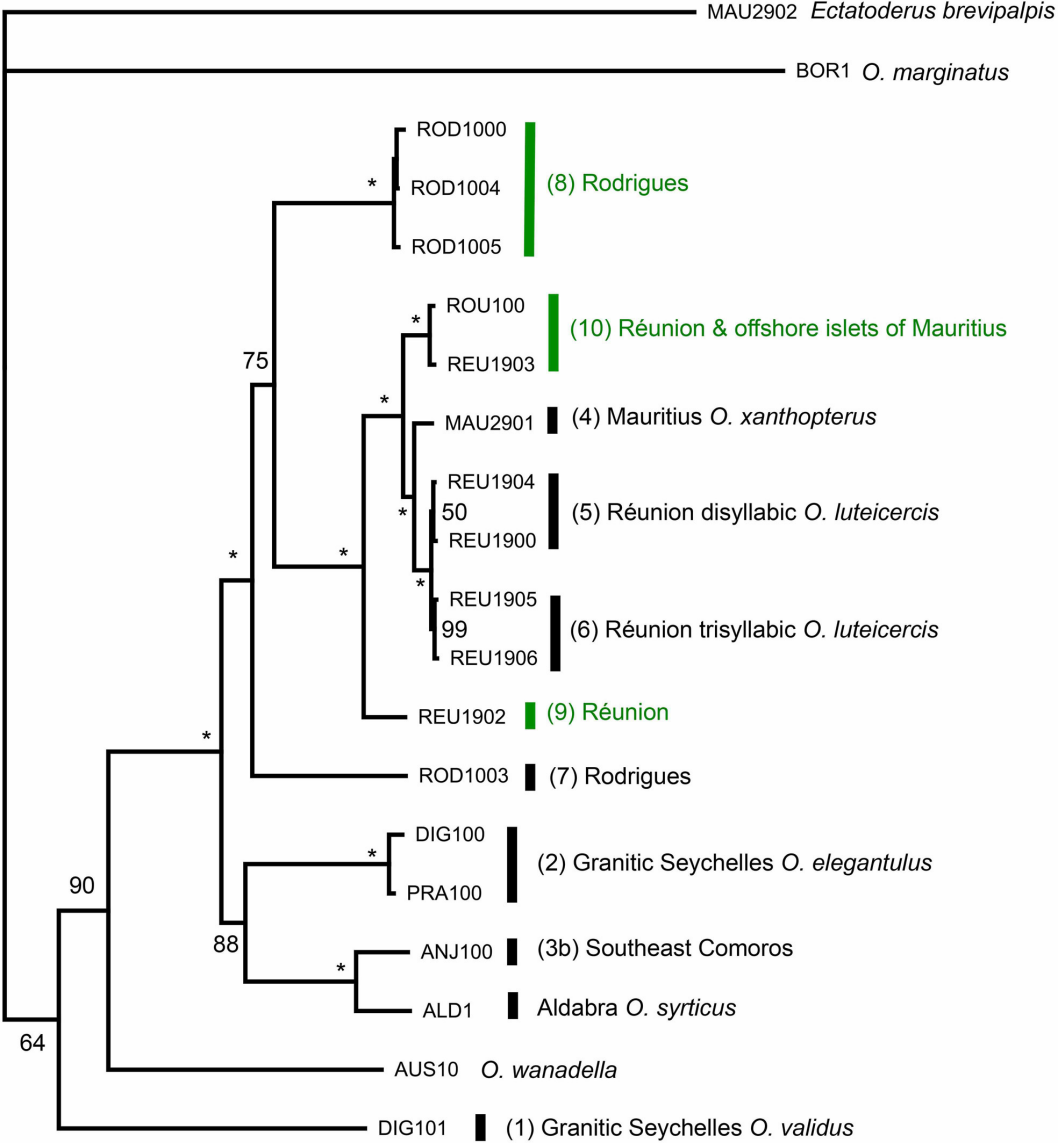
Bayesian analysis of the *Ornebius* combined nuclear-mitochondrial five-gene dataset. Consensus of the last 375,000 trees after 50 million generations, based on the GTR+I+G model. Nodes gaining 100% PP are asterisked, while those gaining 50–99% PP are labelled accordingly. Lineage numbers correspond to song types as listed in Table 1. Lineages labelled in green are associated with *Pandanus*, while those in black are associated with dicots. The islands in which individuals are sampled are coded as in Fig 1; REU, Réunion; MAU, Mauritius; ROD, Rodrigues; ANJ, Anjouan. Islands too small and numerous to label on Fig 1 are as follows. Granitic Seychelles: PRA, Praslin; DIG, La Digue. Aldabra Group: ALD, Aldabra Atoll. Island close to Mauritius: ROU, Round Island.

The four Bayesian analyses yield topology (Fig 5) that is identical to the mitochondrial tree for the full sample set, with respect to supported nodes (≥ 95% Bayesian PP in the mitochondrial tree), as does the MP analysis. The extra data provided by the two nuclear genes increase resolution within the Mascarene clade; *O*. *xanthopterus* and *O*. *luteicercis* are monophyletic (100% PP; Fig 5).

### Biogeographic scenarios, speciation, and the zone of radiation

A null hypothesis of monophyly of the two species of the Grantic Seychelles–*O*. *elegantulus* and *O*. *validus–*consistent with in situ cladogenesis (Scenario 1) can be rejected based on the SH test (P = 0.0014; based on 5-gene dataset). Rather, their phylogenetic placement supports two independent colonisations of the Granitic Seychelles (Scenario 2), the first originating from outside the western Indian Ocean and giving rise to *O*. *validus*, and the second giving rise to *O*. *elegantulus*. By contrast, the monophyly of the Mascarene-endemic clade allows us to reject our default hypothesis of multiple colonisations (Scenario 2) to explain the origin of the six Mascarene-endemic species (*O*. *xanthopterus*, *O*. *luteicercis*, and species 7, 8, 9 and 10). Barring unknown extinctions, all these species descend from a single colonisation of the archipelago, and only in situ cladogenesis can explain their current diversity (Scenario 1). Further, an SH test enables us to reject a scenario involving monophyly of *Pandanus*-associated species (P<0.0001; based on 5-gene dataset). Rather, a minimum of two evolutionary shifts between dicot- and *Pandanus*- association are required to explain the data.

In the case of the Chagos archipelago, the geographic context of species origin is disputable. Unlike the Granitic Seychelles that can be inferred to have been above sea level since separation from India 64 Myr ago, the Chagos was completely submerged during the Quaternary, and likely as recently as 6,500 years ago [38,39]. Therefore, based on island ages, colonisation of the Chagos from the Granitic Seychelles seems more likely than colonisation in the opposite direction. Consequently, for each archipelago we first calculate the number of immigrant, anagenetic and cladogenetic species under the scenario that seems most likely based on island ages, and secondly based on the alternative scenario (Table 2).

**Table 2:** Alternative scenarios for the accumulation of species diversity in western Indian Ocean *Ornebius*.

	Mascarenes	Comoros	Granitic Seychelles	Aldabra Group	Chagos
**Scenario 1**					
Immigrant species	-	-	-	-	*O*. *validus*
Anagenetic species	-	-	*O*. *validus*	*O*. *syrticus*	-
	-	-	*O*. *elegantulus*	-	-
Cladogenetic species	*O*. *xanthopterus*	*O*. *euryxiphus*	-	-	-
	*O*. *luteicercis*	species 3b	-	-	-
	species 7	-	-	-	-
	species 8	-	-	-	-
	species 9	-	-	-	-
	species 10	-	-	-	-
**Scenario 2**					
Immigrant species	-	*-*	*O*. *validus*	-	-
Anagenetic species	-	-	*O*. *elegantulus*	*O*. *syrticus*	*O*. *validus*
Cladogenetic species	*O*. *xanthopterus*	*O*. *euryxiphus*	-	-	-
	*O*. *luteicercis*	species 3b	-	-	-
	species 7	-	-	-	-
	species 8	-	-	-	-
	species 9	-	-	-	-
	species 10	-	-	-	-

Scenario 1 assumes that in the case of *O*. *validus* occurring in two archipelagos, the younger archipelago was colonised from the older one. Thus, the Chagos was recently colonised from the Granitic Seychelles. The alternative, Scenario 2, makes the opposite assumption, with the older archipelago being colonised from the younger one.

**Fig 6:**
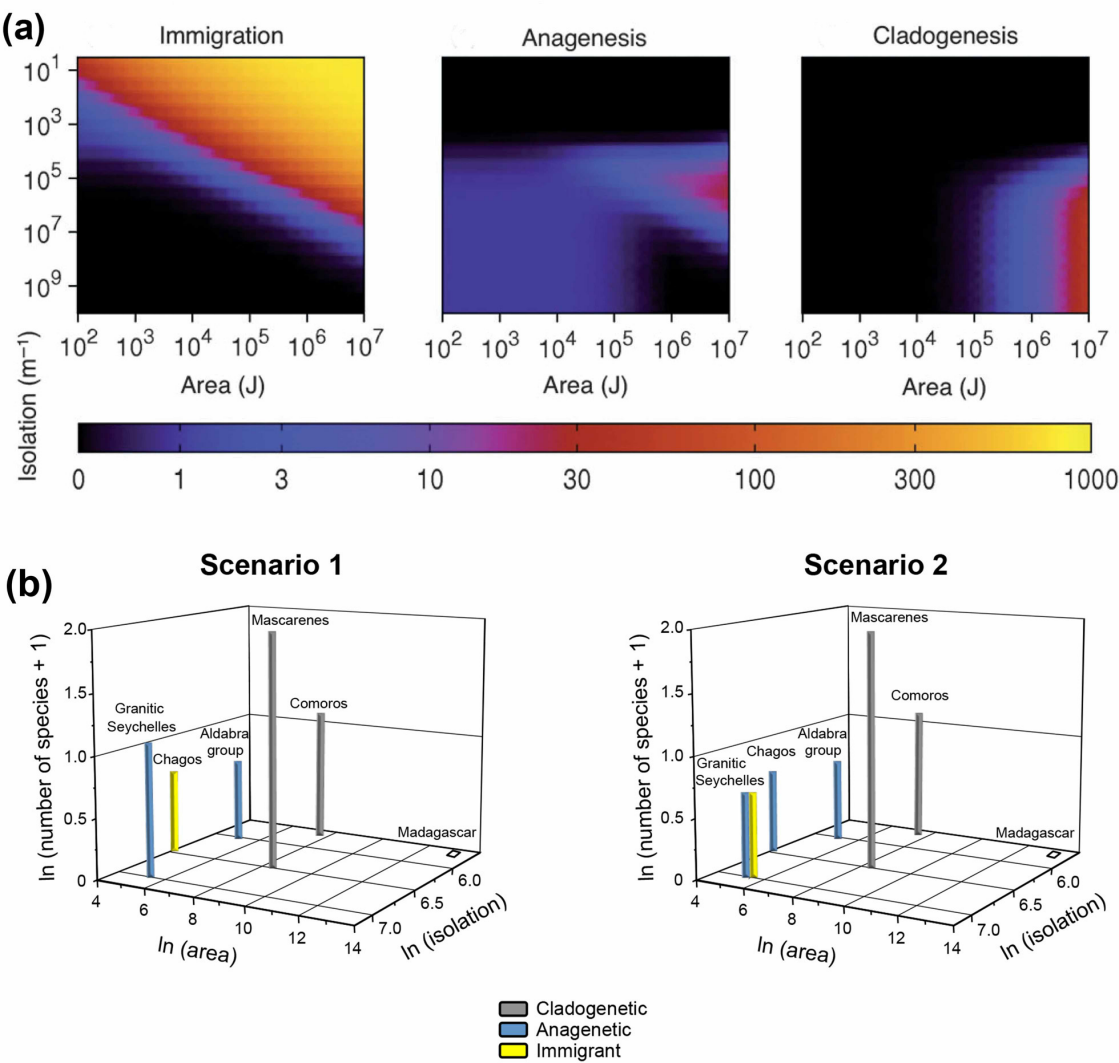
Comparison of patterns of Indian Ocean *Ornebius* species richness with Rosindell and Phillimore’s [10] model. (a) Predictions of the model for the distribution of species richness in immigrant, anagenetic and cladogenetic species with respect to area and isolation. Reprinted from [10], under a CC BY license, with permission from Wiley, original copyright 2011. Scales of species richness are indicated on the horizontal bar. (b) Observed distribution of immigrant, anagenetic and cladogenetic species of *Ornebius* with respect to archipelago area and isolation. *O*. *validus* occurs in two archipelagos. Scenario 1 assumes that the most recently emerged archipelago (Chagos) was colonised from the older one (Granitic Seychelles). The alternative, Scenario 2, makes the opposite assumption, with the older archipelago being colonised from the younger one.

While our phylogeny is consistent with an Australasian or Asian origin for western Indian Ocean *Ornebius*, the monophyly of all western Indian Ocean species except *O*. *validus* suggests that most archipelagos were colonised from within the region. In order to compare archipelago *Ornebius* diversity with Rosindell and Phillimore’s [10] model, archipelago isolation (in km) was therefore measured as the minimum distance from the nearest other landmass. We used the UNEP island directory (http://islands.unep.ch/isldir.htm) and Peake [40] to calculate archipelago area (in km^2^) as the sum of the area of constituent islands. Considering the geography of the western Indian Ocean as a whole, the distribution of immigrant, anagenetic and cladogenetic species do not fit Rosindell & Phillimore’s [10] model (Fig 6). A clear anomaly is the absence of *Ornebius* on the largest landmass, Madagascar. However, the model does seem to have predictive value when excluding Madagascar, and insofar that isolation in all archipelagos can be considered sufficient for anagenetic and cladogenetic speciation. Conforming with the model, cladogenetic species only occur in the two archipelagos that are largest in area (Mascarenes and Comoros).

## Discussion

### Diversity and the zone of radiation

Our acoustic and molecular survey reveals a previously unknown island cricket radiation in the Mascarenes. Following others [10,41] we use ‘island radiation’ in the broadest sense of local cladogenesis, not necessarily involving adaptive radiation nor an increase in diversification rate. Our data imply not only a three-fold increase in the known *Ornebius* species diversity of this archipelago (from two to six species), but also that all this diversity has been formed by in-situ diversification of a single ancestral colonist. The rise in known species number of this archipelago cannot be attributed to taxonomic inflation, considering that each new species is well characterised as an independent evolutionary lineage, none are monophyletic with a single described species, and all are more highly diverged (both from each other and from the described species) than the two described Mascarene species are from each other.

Considering our data from across the western Indian Ocean, we are able to reject our default hypothesis that all cases of double archipelago occupation result from double colonisation from outside the archipelago. While double colonisation is the best explanation for the existence of two species in the Granitic Seychelles, in situ cladogenesis is the only plausible explanation for the six species in the Mascarenes, and two species in the Comoros.

Considering the western Indian Ocean as a whole–Madagascar and surrounding archipelagos–our data do not fit Rosindell and Phillimore’s [10] model (Fig 6). The model does appear to have predictive value when considering the archipelagos surrounding Madagascar alone; the occurrence of cladogenetic species exclusively in the Mascarenes and Comoros (regardless of biogeographic interpretation) is consistent with a zone of cladogenesis restricted to archipelagos that are both relatively isolated and large in size. However, regardless of the biogeographic interpretation used, Madagascar is a significant anomaly. A minimum of six cladogenetic species would be anticipated on Madagascar, considering its size and isolation relative to surrounding islands. Not only do we fail to recover these cladogenetic species, we do not recover *Ornebius* species of any category.

**Fig 7:**
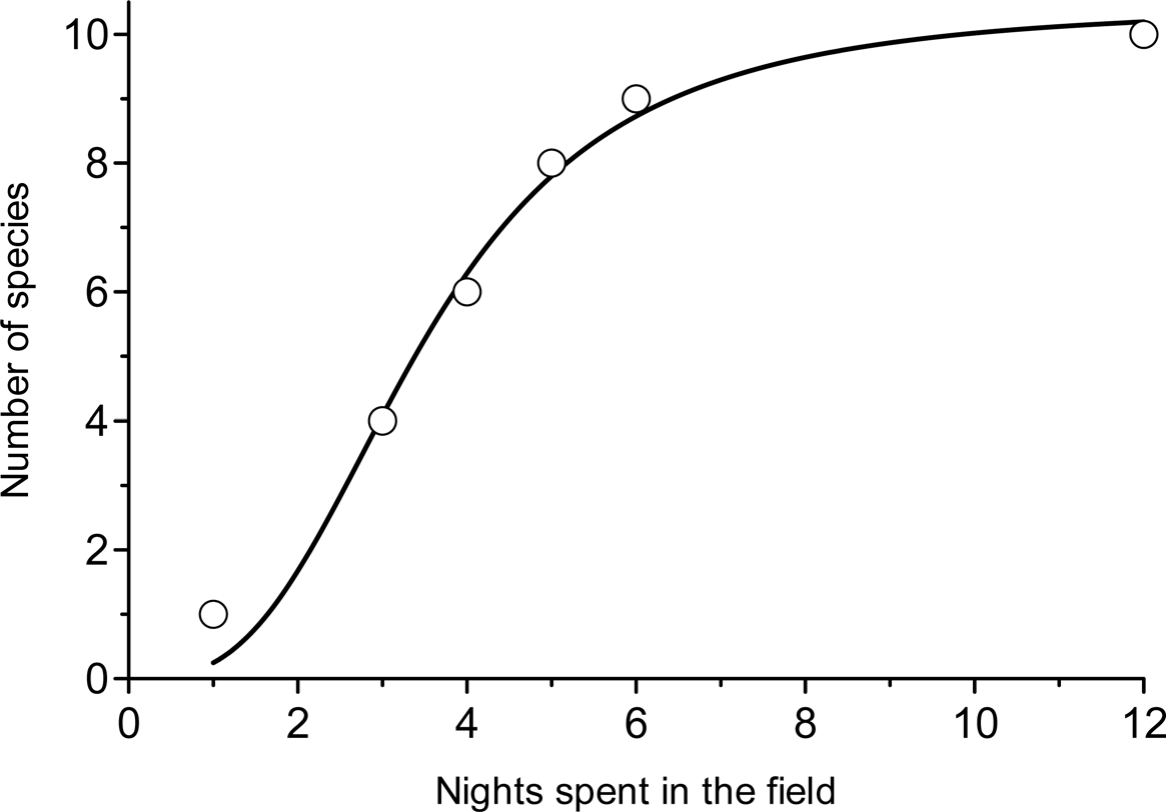
Cumulative distribution of *Ornebius* species encountered as a function of working nights spent in the geographic region occupied by each species (Seychelles; NW Comoros; SE Comoros; Mauritius; Rodrigues; Réunion). Data from all geographic regions are pooled on the same time scale. Data were best fitted with a logistic sigmoid function: N_sp_ = N_spmax_/(1+(t/t_50_)^b^), with the maximal number of species: N_spmax_ = 10.59 ± 0.05; the number of nights required to find half of the species: t_50_ = 3.46 ± 0.02 and the coefficient: b = -2.83 ± 0.05; R = 0.9962. The minimum number of working nights spent in each geographic region was at least 24, corresponding to the time required to reach 99.6% of the expected *Ornebius* species.

While we remain open to the possibility that a small number of *Ornebius* species continue to go undetected on Madagascar, several factors make the non-detection of a radiation of the scale anticipated appear implausible. Although our Madagascar sampling was lower than that for the other islands based on acoustic survey time, it was much higher in terms of museum specimens examined. Over 20 000 unidentified Ensifera of Madagascar origin were available for examination in museums, compared with less than 2 000 specimens (largely identified) from the entire fauna of the surrounding archipelagos. Admittedly, acoustic surveys provide the potential to uncover species undetected by the conventional methods that were usually employed in establishing such collections. Nonetheless, in all of the archipelagos surrounding Madagascar, our species discovery curve (Fig 7) shows that twenty days were sufficient to obtain 99.3% of expected final species diversity. Therefore, were *Ornebius* present at the sites we visited in Madagascar, it is unlikely that we would have failed to recover it in the time spent surveying.

In terms of the biogeographic disjunction posed by lineages present in the Comoros and Mascarenes, but absent in Madagascar, similar cases occur not only in another group of crickets (*Microlandreva*) [42], but also in birds, for which inventories are more reliably complete (*Zosterops* and *Foudia*) [43,44]. All such cases invite questions regarding lineage absence. It is difficult to imagine how terrestrial groups might have dispersed between the Comoros and Mascarenes without colonising Madagascar en route. One explanation is that individuals arrived in Madagascar, but failed to be come established, either as a result of being poorly adapted to the particular ecological conditions present, or as a result of the *Ornebius* niche already being occupied by other species. Such parameters are not taken into account in Rosindell and Phillimore’s [10] model, which is ecologically neutral. While *Pandanus* is abundant in Madagascar, with much higher species diversity than in the Mascarenes (84 versus 23 endemic species, respectively), it can be imagined that a long period of independent evolution in small oceanic island populations rendered colonist *Ornebius* lineages ill-suited to the continental island conditions of Madagascar. Although we are unable to identify species competitively excluding *Ornebius* from Madagascar, the absence from Madagascar of other orthopteran species widespread in Africa and surrounding islands (*Afroxyrrhepes procera*: Africa and Comoros, *Diabolocatantops axillaris saucius*: Africa, Comoros, Aldabra and Iles Eparses) [45,46], lends some support to the idea of competitive exclusion.

As an alternative explanation for the biogeographic disjunction, a wave of recent (yet pre-human) extinction in Madagascar has been invoked for birds on numerous grounds [47,48]. Goodman and Jungers [49] suggest that widespread megafaunal extinction may have been linked to natural environmental change. This includes changes in climate linked to global glacial-interglacial cycling, which may have had more extreme effects on Madagascar than on the buffered climates of surrounding oceanic archipelagos. It also includes climatic change linked to volcanism on Madagascar (from approximately 14 000 to 8000 BP; [49]). Given the putatively pervasive effects of such environmental change on the vertebrate fauna, it seems plausible that invertebrate groups such as *Ornebius* were also affected.

### Cladogenesis in the Mascarenes

Like the other *Ornebius* species in the western Indian Ocean, three of the Mascarene species are associated with dicotyledonous plants (*O*. *xanthopterus*, *O*. *luteicercis*, and species 7). The other three (species 8, 9 and 10) are associated with screw pines (*Pandanus*). This association appears to be unique to the Mascarene species, being absent from the other western Indian Ocean islands based on our surveys, and being unreported for all other *Ornebius* species worldwide [50,51; Sigfrid Ingrish, David Rentz, pers. comm.].

**Fig 8:**
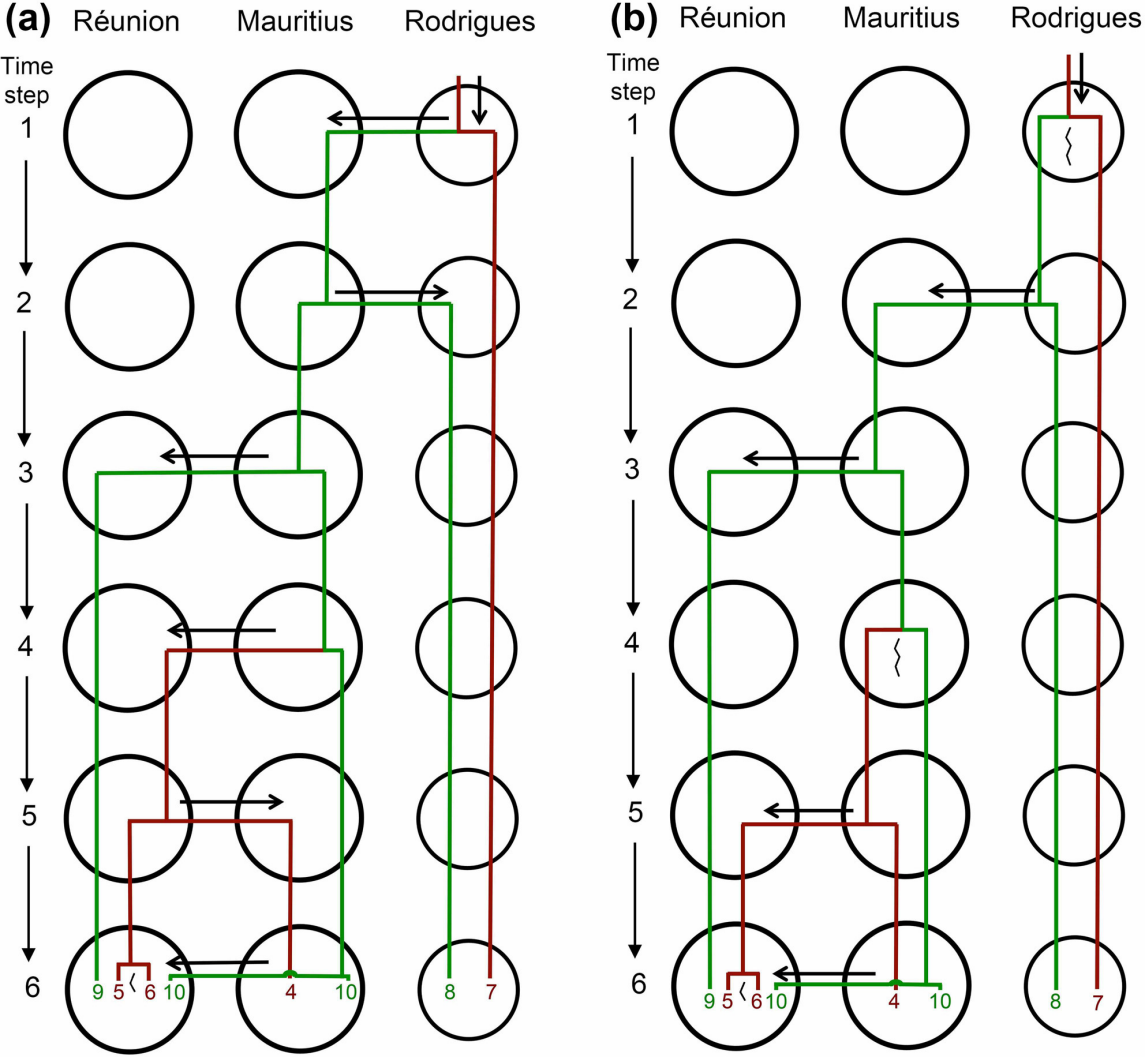
Mascarene *Ornebius* speciation scenarios. (a) Scenario invoking inter-island speciation alone. (b) Scenario invoking intra- as well as inter- island speciation. Lineages associated with dicotyledonous plants are in brown, while those associated with *Pandanus* are in green. Lineage numbers correspond to song types in Table 1. Long jagged lines indicate intra-island speciation. Short jagged lines indicate intra-island divergence. Time step 6 represents the present day, in which song types 5 and 6 show weak genetic divergence, while song type 10 does not show any divergence between Réunion and the offshore islets of Mauritius. For simplicity, we depict song type 10 as undergoing present-day dispersal to Réunion, but acknowledge that in reality dispersal (and associated gene flow) may be bidirectional and ongoing. Note that these are the simplest scenarios ignoring extinction. More complex scenarios are also possible.

Contrary to intuition, our phylogeny allows us to reject a scenario of a single evolutionary shift in ecology between dicot-association and *Pandanus*-association followed by inter-island colonisation and diversification of the descendant lineages. Rather, a minimum of two shifts between dicot- and *Pandanus*- association are required to explain our data. A scenario in which speciation and ecological divergence occurs purely at the inter-island scale is conceivable, being consistent with this requirement. Such a scenario is further consistent with the phylogeny (none of the single island species assemblages being monophyletic), and current species ecologies and distributions (Fig 8A). Despite the simplicity of this speciation scenario, a number of factors make alternative scenarios invoking intra-island speciation (either driven by, or associated with, ecological divergence) appear at least as likely (Fig 8B being one among a multitude of possibilities). First, that *Ornebius* populations can diverge at the intra-island scale is supported by the existence of two geographically distinct song groups of *O*. *luteicercis* on the island of Réunion, exhibiting a small degree of genetic and ecological divergence. Second, despite the simplicity of speciation by inter-island colonisation, the scenario is biogeographically complicated (Fig 8A) when compared with one that allows intra-island speciation (Fig 8B), with more inter-island colonisation events required. Third, the habitat diversity and topographic complexity of Réunion today, and of Mauritius and Rodrigues in the geological past [52–54], make intra-island barriers to gene flow (whether permeable or impermeable) easy to conceive for flightless crickets. Fourth, the abundance of intra-island speciation in crickets of the Hawaiian and Caribbean islands, as supported by the monophyly of species endemic to the same island, is evidence of the feasibility of speciation in crickets at this spatial scale.

Many different scenarios invoking intra-island speciation can be envisaged. A scenario of a single shift to *Pandanus*-association followed by a single back-shift to dicot-association involves the fewest ecological changes and is therefore the one we choose to illustrate (Fig 8B). Insight into the processes leading to intra-island divergence and ultimately speciation might be gained by considering the two populations of *O*. *luteicercis* differing in song pattern and exhibiting a small degree of genetic divergence. Intra-island geographic segregation appears to be key in song divergence, with the disyllabic population only known from the northwest quarter of the island—the Cirque de Mafate at mid-altitude (≤ 800 m) down to the sea, and the trisyllabic population only from the remaining southeast portion of the island within the same altitudinal range (0–800 m). Currently, these two populations are not known to come into contact, and it is therefore conceivable that they diverged in full allopatry with a total absence of gene flow. However, the barrier between the two at low altitude is currently landscape that has been human-modified, and it seems likely that the two populations may have been in contact prior to human arrival, with gene flow possible.

In the Hawaiian islands, many *Laupala* species are sympatric or parapatric and cannot be distinguished on the basis of ecological characteristics. It is suggested that correlated evolution of song and song preference could be a mechanism that promotes assortative mating among populations, thus reducing gene flow and leading to speciation [14,55,56]. However, what causes initial divergence in song is unclear; reproductive character displacement across part of a species range as a result of interactions with parapatric congeners has been suggested, but remains to be tested [56]. In *O*. *luteicercis*, our observations based on the small number of individuals encountered are suggestive of ecological differences between populations, with the disyllabic population occurring closer to the ground than the trisyllabic population. That both ecological and song differences occur at an intra-specific scale between populations apparently undergoing divergence (di- and tri- syllabic *O*. *luteicercis*), rather than being restricted to species that are already highly diverged (dicot- and *Pandanus*-associated species), is suggestive of a speciation scenario in which ecological and mating traits diverge hand-in-hand [57–59]. Further genetic and ecological study of the two *O*. *luteicercis* populations may shed light on the mode(s) of speciation of the Mascarene radiation as a whole.

Regardless of which scenario of speciation is the correct one, it seems that the occurrence of the most diverse *Ornebius* archipelago radiation in the Mascarenes cannot be explained by the presence of *Pandanus* alone, since *Pandanus* is also present in all the other archipelagos under consideration. Rather, isolation of the archipelago appears to have been key. A low rate of immigration on remote islands may increase ecological opportunity (i.e. more ‘open’ niches are available, and for longer periods [60]). It may also lower the rate of turnover (immigration-extinction), affording anagenetic species more time to diverge in situ [4,10,61]. Both factors might have been important in promoting cladogenesis in Mascarene *Ornebius*.

In conclusion, our results suggest that despite the importance of island size and isolation in determining where a radiation is observed, environmental differences between islands may be at least as significant. Our surveys demonstrate how easily tropical invertebrate diversity can be missed, even in archipelagos that are heavily populated and otherwise well known. We suggest that surveying for acoustic diversity, followed by DNA sequencing of the populations uncovered, may be a useful way of accelerating future biodiversity discovery in tropical island crickets.

## Supporting Information

S1 File Data used for song classification. (XLSX)

S1 Table Parameters used for *Ornebius* song classification. (PDF)
