# Correction: Predicting Where a Radiation Will Occur: Acoustic and Molecular Surveys Reveal Overlooked Diversity in Indian Ocean Island Crickets (Mogoplistinae: *Ornebius*)

**DOI:** 10.1371/journal.pone.0153504

**Published:** 2016-04-14

**Authors:** Ben H. Warren, Rémy Baudin, Antoine Franck, Sylvain Hugel, Dominique Strasberg

The Table 2 footnotes are incorrectly typeset in the body of the article, as paragraph 3 of the “Biogeographic scenarios, speciation, and the zone of radiation” subsection of the Results section. This can be found on page 12 of the article PDF. The footnotes should read: “Scenario 1 assumes that in the case of *O*. *validus* occurring in two archipelagos, the younger archipelago was colonised from the older one. Thus, the Chagos was recently colonised from the Granitic Seychelles. The alternative, Scenario 2, makes the opposite assumption, with the older archipelago being colonised from the younger one.” Please view the correct Table 2 with its footnotes here.

**Table 2:** Alternative scenarios for the accumulation of species diversity in western Indian Ocean *Ornebius*.

	Mascarenes	Comoros	Granitic Seychelles	Aldabra Group	Chagos
**Scenario 1**					
Immigrant species	-	-	-	-	*O*. *validus*
Anagenetic species	-	-	*O*. *validus*	*O*. *syrticus*	-
	-	-	*O*. *elegantulus*	-	-
Cladogenetic species	*O*. *xanthopterus*	*O*. *euryxiphus*	-	-	-
	*O*. *luteicercis*	species 3b	-	-	-
	species 7	-	-	-	-
	species 8	-	-	-	-
	species 9	-	-	-	-
	species 10	-	-	-	-
**Scenario 2**					
Immigrant species	-	*-*	*O*. *validus*	-	-
Anagenetic species	-	-	*O*. *elegantulus*	*O*. *syrticus*	*O*. *validus*
Cladogenetic species	*O*. *xanthopterus*	*O*. *euryxiphus*	-	-	-
	*O*. *luteicercis*	species 3b	-	-	-
	species 7	-	-	-	-
	species 8	-	-	-	-
	species 9	-	-	-	-
	species 10	-	-	-	-

Scenario 1 assumes that in the case of *O*. *validus* occurring in two archipelagos, the younger archipelago was colonised from the older one. Thus, the Chagos was recently colonised from the Granitic Seychelles. The alternative, Scenario 2, makes the opposite assumption, with the older archipelago being colonised from the younger one.
