# Supplementary material for: Predicting Where a Radiation Will Occur: Acoustic and Molecular Surveys Reveal Overlooked Diversity in Indian Ocean Island Crickets (Mogoplistinae: Ornebius)
Source: PLoS One. 2016 Feb 12;11(2):e0148971. doi: 10.1371/journal.pone.0148971 (PMC4752360; doi:10.1371/journal.pone.0148971)
Supplement: S1 Table — (PDF) [file pone.0148971.s002.pdf]

S1 Table. Parameters used for *Ornebius* song classification

Parameter	Description
Syllables	Number of syllables per sentence (1, 2 or 3)
Rate1	Inverse of the period of the 1st syllable (P1)
Rate2/Rate1	Inverse of the period of the 2nd syllable (P2), normalized by Rate1 (=1 in monosyllabic pattern)
Rate3/Rate1	Inverse of the period of the 3rd syllable (P3), normalized by Rate1 (=1 in monosyllabic and disyllabic patterns)
Fundamental	Frequency of the song (corresponds to the 1st harmonic)
Sentence period	Total duration of a cycle (either mono-, di- or trisyllabic)
Rate1/frequency	Ratio of Rate 1 and the frequency of the song
Rate2/frequency	Ratio of Rate 2 and the frequency of the song
Rate3/frequency	Ratio of Rate 3 and the frequency of the song
